# Association of Genetic and Environmental Factors with Non-Alcoholic Fatty Liver Disease in a Chinese Han Population

**DOI:** 10.3390/ijerph17145217

**Published:** 2020-07-20

**Authors:** Zheng Li, Cheng-Yin Ye, Li Wang, Jin-Mei Li, Lei Yang

**Affiliations:** Medical School, Hangzhou Normal University, Hangzhou 310000, China; lizheng@stu.hznu.edu.cn (Z.L.); yechengyin@hznu.edu.cn (C.-Y.Y.); 2019111012034@stu.hznu.edu.cn (L.W.); lijinmei@stu.hznu.edu.cn (J.-M.L.)

**Keywords:** NAFLD, elastic net, single-nucleotide polymorphism, environmental factors

## Abstract

Lifestyle choices such as the intake of sweets, history of diseases, and genetic variants seem to play a role in the pathogenesis of non-alcoholic fatty liver disease (NAFLD). To explore which genetic and environmental factors are associated with NAFLD in a Chinese Han population, we conducted this study. We collected the medical reports, lifestyle details, and blood samples of individuals and used the polymerase chain reaction-ligase detection reaction method to genotype the single-nucleotide polymorphism (SNPs) from the 2113 eligible people. The GG genotype of the additive model of rs7493 in the PON2, the CC genotype of the additive and recessive models of rs7593130 in the ADCY3, together with dyslipidemia, regular intake of egg and sweets and hypertension, increased the risk of NAFLD (adjusted OR > 1, *p* < 0.05). The TT genotype of the additive and dominant models of rs11583680 in the PCSK9, together with the regular intake of vegetable, reduced the risk of NAFLD (adjusted OR < 1, *p* < 0.05). In addition, interactions between some variables were found. Eventually, we identified three SNPs and six environmental factors associated with NAFLD. These results provide the theoretical basis for gene and other risk factors screening to prevent NAFLD.

## 1. Introduction

With the increasing prevalence of metabolic syndromes such as obesity as well as a spectrum of liver disorders, non-alcoholic fatty liver disease (NAFLD) has become a very frequent disease [1]. An epidemiological study involving more than 8 million subjects from 22 countries showed that the global prevalence of NAFLD was 25.2% [2], at the same time, according to a recent meta-analysis, an estimated one in four adults in the world suffer from NAFLD [2,3]. The estimated annual impact of the disease burden on the US economy is $103 billion and the combined economic impact on the UK, Germany, France, and Italy is €35 billion [4].

The mechanisms that lead to NAFLD are unclear to date. For a long time, fatty liver was considered as a complex disease influenced by genetic and environmental factors. Among the many proposed mechanisms, insulin resistance seems to be pivotal in the pathogenesis of both NAFLD and type 2 diabetes [5]. In addition, earlier studies have shown that the presence of sedentarism, dyslipidemia, hypertriglyceridemia, obesity, hypertension, and older age seem to play a role in the pathogenesis of NAFLD [2,5,6,7,8,9,10,11].

Gene polymorphisms have also been proposed as one of the contributing factors to the pathogenesis of NAFLD. A variety of genome-wide associations have emerged in recent years, which have enriched our understanding of the genetic basis of NAFLD [12,13]. For example, the transmembrane 6 superfamily member 2, encoding a glutamate with lysine change at codon 167 (E167K) has been performed to elucidate the potential role of candidate genes in the pathogenesis and progression of NAFLD [14].

The main genes involved in our research are Paraoxonase 2 (PON2), adenylate cyclase 3 (ADCY3), Pro-protein convertase subtilisin-kexin type 9 (PCSK9), and glucokinase regulator (GCKR). Paraoxonase 2 (PON2), a member of the paraoxonase gene family, was wildly expressed and localized to the nuclear envelope, endoplasmic reticulum, and mitochondria, showing a causal relationship between mitochondrial dysfunction and obesity in mice and humans [15,16]. Similarly, recent studies have identified a variant in ADCY3, which is widely distributed, high in subcutaneous and visceral adipose tissue, and associated with a significantly increased risk of obesity, and loss of function mutations in ADCY3 in mice leads to a severe obesity phenotype [17,18,19].

Pro-protein convertase subtilisin-kexin type 9 (PCSK9) is an enzyme that promotes low-density lipoprotein receptor (LDLR) degradation upon activation [20]. Recent studies have identified that liver fat accumulation is associated with circulating PCSK9 [21,22]. Variants with moderate effect size in the glucokinase regulator (GCKR) have also been shown to have a significant contribution to NAFLD [23]. The GCKR mutation (rs1260326) encoding for the P446L protein variant is a common missense deficiency and appears to represent a causal variation associated with hepatic fat accumulation [24].

In this study, we explore a new possibility about the cause of NAFLD. Furthermore, we comprehensively analyzed the potential interactions between genes, physiological indices, biochemical indicators, behavioral factors, and NAFLD. Finally, we constructed a model by elastic net that included genes and other environmental factors to identify variables strongly associated with NAFLD rather than to predict the occurrence of NAFLD. Taken together, our results show that the variants of the PON2, ADCY3, PCSK9, and GCKR, together with dyslipidemia, regular intake of eggs and sweets, and hypertension, increased the risk of NAFLD. At the same time, interactions between some variables were found in our study; this provides the theoretical basis for gene and other risk factors screening to prevent NAFLD.

## 2. Materials and Methods

### 2.1. Subjects

A total of 2323 subjects were randomly selected from 4 towns and townships in a district of Ningbo, Zhejiang Province from those who underwent physical examination from April 2013 to July 2016. We collected individual medical reports, lifestyle details, and blood samples. Then, we used the polymerase chain reaction-ligase detection reaction method to perform genotyping for single-nucleotide polymorphisms (SNPs). Interviews were performed in August 2016 to determine the subjects’ incidence of NAFLD. The case group included 327 patients (as evaluated by the same experienced operator) diagnosed with NAFLD between April 2013 and August 2016. All subjects were unrelated and >40 years of age. A total of 2113 people qualified for the study. Biomarkers, such asalanine aminotransferase (ALT) or aspartate aminotransferase (AST), and imaging techniques, such as ultrasonography, magnetic resonance imaging (MRI), computerized Tomographic (CT), scanning or transient elastography, were employed in diagnosis of NAFLD [25,26]. We excluded patients diagnosed with NAFLD in April 2013. At the same time, patients with viral hepatitis, autoimmune liver disease, and drug hepatotoxicity were excluded. All participants signed informed consent forms and the study was approved by the Medical Ethics Committee of Hangzhou Normal University (No. 2013020).

### 2.2. Demographic Information and Epidemiological Investigation

Demographic variables collected in this study included sex, age, occupation, education level, medical history, as well as lifestyle information such as smoking, drinking, diet, and physical activity intensity. A dietary behavior survey was administered as a semiquantitative questionnaire to determine the food type, intake, and frequency. The main lifestyle variables were defined as follows. (1) Diet: “drink milk” and “drink soymilk” were defined as maintaining a certain amount of milk or soymilk intake every day, whereas “no milk intake habit” was defined as “not drinking”. An average intake of fried food of less than 1 time per week was defined as “no fried food”; those who ate less than one sweet treat per week were defined as “not eating sweets”. (2) Smoking: smoking behavior was defined as smoking at least one cigarette per day for at least 1 year. (3) Physical activity classification: “there is little physical activity, e.g., desk workers such as secretaries” was defined as “sedentary”; “Light physical activity” was defined as “office work, repair of electrical clocks and watches, sales clerks, hotel services, chemical laboratory operations, lectures, etc.”; “Students’ daily activities, motor vehicle driving, electrical installation, lathe operation, metal cutting, etc.” was defined as “moderate physical activity”; “Non-mechanized agricultural labor, steelmaking, dancing, sports movement, loading and unloading, mining, loading and unloading cargo, construction workers, etc.” was defined as “heavy physical activity”.

Anthropometric data, including systolic blood pressure (SBP), diastolic blood pressure (DBP), weight, waist circumference, BMI, total cholesterol (TC), triglycerides (TG), high-density lipoprotein-cholesterol (HDL-C), and low-density lipoprotein-cholesterol (LDL-C) levels, were evaluated by professional medical examinations according to standard protocols.

### 2.3. Isolation of Genomic DNA

Blood samples were collected from the antecubital vein after the subjects had fasted for ≥8 h. Part of the collected samples was used to examine biochemical indicators such as serum lipid levels, whereas the other part was transferred into a test tube containing anticoagulant solution to extract DNA. DNA was extracted using Tiangen Blood Genomic DNA extraction kits (Tiangen Biotech, Beijing, China), we use a PCR-LDR method to genotyping analysis as we described in detail in our previous study [27].

### 2.4. SNP Selection and Genotyping

We mainly used the PubMed, Kyoto Encyclopedia of Genes and Genomes, and GeneCard databases to select single-nucleotide polymorphism (SNPs). The specific screening process was as follows. (1) Literature related to gene polymorphism, dyslipidemia, fatty liver, and atherosclerosis were searched on NCB-PubMed, and SNPs were screened; (2) GeneView information was obtained for relevant SNPs from the GeneCards database and NCBI database, and then, missense mutations, 3′ untranslated region (3′ UTR), 5′ UTR, or transcription factor-binding sites were selected; (3) The minor allele frequency (MAF) of SNPs in the Chinese population was detected from the HapMap database for the international human genome, and SNP sites with MAF values greater than 0.05 were screened; (4) Haploview software was used to conduct linkage imbalance analysis on all selected sites, and tagSNP was selected with r^2^ ≥ 0.8 as the standard.

This process identified 102 SNPs. Information regarding all SNP loci is shown in Table S1.

### 2.5. Statistical Analysis

Statistical analysis was conducted with SPSS 24.0 software (SPSS Inc., Chicago, IL, USA) and glmnet package of RStudio (Version 1.1.456. RStudio, Boston, MA, USA; http://www.rstudio.org/) [28]. The chi-squared test, Fisher exact test (for categorical variables), t test, and Wilcoxon rank sum test (for continuous variables) were used to evaluate demographic characteristics and SNP genotypes. The odd ratios (ORs) and 95% confidence intervals (CIs) determined by logistic regression analysis were used to analyze the associations between genetic models, lifestyles and the risk of NAFLD. To shrink the model to reduce overfitting and covariate correlation and feature selection, we used elastic net regularization [29]. The results show that this method is superior to other analysis methods when the number of feature sets is much larger than the number of cases, which makes use of the advantages of both least absolute shrinkage and selection operator (LASSO) and ridge [30]. The logistic-regression model based on the 102 SNP feature selection and model based on SNP/lifestyle features were separately developed on an elastic net. A gene score was calculated for each person via the elastic net of 4 selected SNPs weighted by their respective coefficients. The gene scores were combined with 31 environmental variables and 9 variables were screened out, including gene scores with nonzero coefficients as determined by logistic regression. Finally, receiver operating characteristic (ROC) curves were plotted to assess the efficiency of the model. Excel software was used to calculate the relative excess risk due to interaction (RERI), OR, and 95%CI as described by Knol et al. [31]. Haploview, plink, and g-plink were used to calculate the *p*-values of Hardy–Weinberg equilibrium. In all analyses, *p*-values < 0.05 were considered to indicate a statistically significant difference.

## 3. Results

### 3.1. General Characteristics

The prevalence of NAFLD in the study population is 15%, which is similar to that reported by epidemiologists (10–30%) [4]. A summary of their demographic characteristics, such as age, sex, weight, BMI, waistline, etc., is shown in Table 1. There were significant differences in weight; Body Mass Index (BMI); waistline; systolic blood pressure (SBP); diastolic blood pressure (DBP); total cholesterol (TC); triglycerides (TG); high-density lipoprotein-cholesterol (HDL-C); low-density lipoprotein-cholesterol (LDL-C); the history of diabetes and smoking; the intake of vegetables, fruit, milk, soybean milk, egg, meat, fried foods, sweets, and salt between the case and control groups (*p* < 0.05) (Table 1). All studied SNPs in the control subjects were in Hardy–Weinberg equilibrium (*p* > 0.05) (Table S1).

### 3.2. Gene-Based Model: SNPs Associated with NAFLD

**Figure 1:**
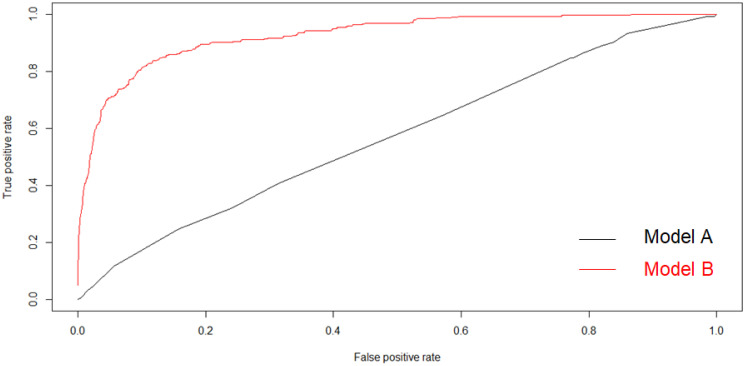
Area under the receiver operating characteristic curve (AUC) of model A and model B.

Elastic net penalization allows for variable selection by shrinking the coefficients of the variables not related to the response to zero. Initially, 102 SNPs were reduced to four potential predictors in 2113 people, and were features with nonzero coefficients in the elastic net model (Model A). The four potential SNPs were rs7493 in PON2, rs7593130 in ADCY3, rs1260326 in GCKR, and rs11583680 in PCSK9. The area under the receiver operating characteristic curve (AUC) for model A was 0.57 (Figure 1). The black line represents model A, which was generated from SNP features using elastic net regression.

Table 2 is the association between the 4 SNPs and environmental factors with NAFLD, which was examined under each gene model. Without adjustment, the dominant model of rs7493 and rs11583680 and the recessive model of rs7593130 were found to be significantly associated with NAFLD (unadjusted OR = 1.31, 95%CI = 1.03–1.68, *p* = 0.026; unadjusted OR = 0.61, 95%CI = 0.447–0.83, *p* = 0.002; unadjusted OR = 1.54, 95%CI = 1.14–2.07, *p* = 0.005). Subjects carrying the CC genotype in the recessive model of rs7593130, GG + CG genotype in the dominant model of rs7493, and CC genotype in the dominant model of rs11583680 showed a lower risk of NAFLD than those with the CT + TT genotype, CC genotype, TT + CT genotype, and CC genotype (Table 2). This means that the people who carry the GG genotype of rs7493, the CC genotype of rs7593130, the TT genotype of rs1260326, and the TT genotype of rs1260326 had a higher risk of NAFLD.

### 3.3. All Covariance-Based Model

Considering that model A only focused on the influence of genes on NAFLD, we recreated model B, which included genetic characteristics and physiological, biochemical, and lifestyle indicators to identify factors related to NAFLD. When 102 SNPs were reduced to four potential predictors, the features of the four SNPs were presented in the gene score calculation formula by elastic net. A gene score was calculated for every person by linear combination of the selected features weighted by their respective coefficients. The gene score was combined with 31 lifestyle variables, and seven variables with gene scores were screened out by logistic regression (Model B). The AUC for model B was 0.86 (Figure 1). The seven variables were gene score; dyslipidemia; sex; SBP; and the intake of eggs, sweets, and vegetables. Except for sex, the other six variables had statistical significance for the occurrence of NAFLD (Table 3). Table S2 is the Elastic net regularization feature selection for gene score and lifestyles.

After adjusting for these six variables, the recessive models of rs7593130 and dominant models of rs74932 and rs11583680 were still significantly associated with NAFLD (*p* < 0.05) (Table 2). In the additive models, the CC genotype of rs7493 and rs1260326, TT genotype of rs7593130 and rs11583680, still reduced the risk of NAFLD (Table 2).

### 3.4. Interactions between Gene Polymorphism and Other Covariance Estimators for the Risk of NAFLD

Except for a few disease cases purely associated with genetic disease or environmental factors, the vast majority of diseases is the result of a combination of genetic and environmental factors, especially for complex traits of chronic diseases, in which the interaction of genetic and environmental factors plays a very important role. Therefore, we further focus on the contribution of genetic and lifestyle interactions in the development of NAFLD using crossover analysis. As neither the dominant nor the recessive model in rs1260326 was statistically significant for the occurrence of the disease, we only discussed the interaction between the other three SNPs and environmental factors.

Table 4, Tables S3 and S4, respectively, show the effects of the interaction between three SNPs and the intake of vegetables, the intake of egg, and the intake of dessert on NAFLD. Basically, the three tables show that individuals with poor lifestyle were at higher risk of NAFLD compared with individuals in the same genetic risk category but with an ideal lifestyle. For example, in rs11583680, rs7593130, and rs7493, compared to in subjects who eat plenty vegetables carrying the non-risk genotype, those without consumption of plenty vegetables who carried the non-risk were at a lower risk of NAFLD (OR = 7.25, 95%CI = 3.90–13.48, *p* < 0.001; OR = 8.08, 95%CI = 6.59–11.74, *p* < 0.001; OR = 19.84, 95%CI = 7.07–13.68, *p* < 0.001) (Table 4). The same results can be seen in Tables S3 and S4. Importantly, the studies also showed individuals with ideal lifestyle and high genetic risk were at a 2–3-fold higher risk of developing NAFLD compared with individuals with an ideal lifestyle but low genetic risk. For example, in rs11583680, rs7593130, and rs7493, compared to individuals with low genetic risk and proper intake of eggs, people with proper intake of eggs and high genetic risk were at a higher risk of NAFLD (OR = 34.15, 95%CI = 17.69–65.94, *p* < 0.001; OR = 29.03, 95%CI = 17.43–48.35, *p* < 0.001; OR = 12.99, 95%CI = 8.77–19.22, *p* < 0.001) (Table S3). The difference was that the interactions between rs11583680, rs7593130, and vegetable consumption were statistically significant (RERI = 4.91, 95%CI = 0.66–9.17, *p* = 0.024; RERI = 7.55, 95%CI = 0.13–14.97, *p* = 0.046), while the interactions between other variables were not (Table 4, Tables S3 and S4). Although an interaction between the three SNPs and other lifestyles was not found (*p*-values of RERI > 0.05), there was a cumulative effect in each model. For example, in rs11583680, within the strata of CC, people who eat inadequate vegetables had a higher risk of NAFLD than those who eat adequate vegetables; moreover, in people who had inadequate intake of vegetables, people who carry the risk allele have a higher risk (OR = 9.49, 95%CI = 7.12–12.64, *p* < 0.001; OR = 1.31, 95%CI = 1.04–1.66, *p* = 0.022) (Table 4). The same results can be seen in other SNPS and lifestyles (Table 4, Tables S3 and S4).

Table 5 shows the effects of the interaction between dyslipidemia and other environmental factors on NAFLD. A positive interaction between dyslipidemia and vegetable consumption was found. The same results were found in egg consumption and sweet consumption (RERI = 13.97, 95%CI = 4.87–23.12, *p* = 0.002; RERI = 17.47, 95%CI = 4.55–30.39, *p* = 0.008; RERI = 12.08, 95%CI = 1.77–22.38, *p* = 0.022). Although an interaction between the hypertension and dyslipidemia was not found (*p*-values of RERI > 0.05), within the strata of hypertension, people with a history of dyslipidemia had a higher risk of NAFLD than those without a history of dyslipidemia (OR = 1.84, 95%CI = 1.27–2.66, *p* = 0.001) (Table 5).

In addition, we also explored the effects of the interaction between gene polymorphism, hypertension, and dyslipidemia on NAFLD. Tables S5 and S6 show the results. Similar to the results above, individuals with hypertension or dyslipidemia were at higher risk of NAFLD compared with individuals in the same genetic risk category but without hypertension or dyslipidemia.

## 4. Discussion

As is well known, LASSO is famous for its performance with variable selection, and ridge is suitable for multicollinearity [30]. Although both of them can select variables, the elastic mesh performs better than the LASSO when the data is collinear. In this study, there are hundreds of variables, which cannot remove the multicollinearity problem, so we choose the elastic network method. When 102 SNPs were reduced to four potential predictors by elastic net, a gene score presenting the features of the four SNPs was calculated for every person. Then, a logistic regression model including 31 environmental factors and gene score was also employed to explain the relationship between candidate features and NAFLD. The model, which makes use of easily accessible metrics, can serve as a more convenient biomarker for explaining NAFLD.

The identification of risk alleles is useful because if the involved genes and their functions are known, this information can be used to develop d prevention, treatment, prognosis prediction, and/or curative methods for the disease. Genetic analysis allows us to better understand the biological mechanisms of NAFLD. We found that the minor allele (“C”) of rs7493 in PON2, the minor allele (“T”) of rs7593130 in ADCY3, and the minor allele (“C”) of rs11583680 in PCSK9 were associated with a reduced risk of NAFLD in the Chinese population, whereas the minor allele (“C”) of rs1260326 in GCKR was associated with a reduced risk of NAFLD.

The mechanism by which PON2 affects NAFLD is unknown, but from previous studies, we can infer two possibilities. Studies have demonstrated that PON2 deficiency is associated with the drop of energy expenditure and oxygen consumption, which may be the underlying cause of obesity [19,32], while it is well known that obesity is an important risk factor for NAFLD. Results from various laboratories suggested that PON2 deficiency leads to mitochondrial dysfunction and an increase in mitochondrial oxidative stress [15,16], which may be part of the reason for the increase in LDL oxidative modification [15,33]. Furthermore, it is associated with the occurrence of NAFLD. Either way, however, further research is needed. Similarly, adenylate cyclase 3 (ADCY3) mutations have been implicated in obesity [17,18,34]. In addition, genetic studies have indicated that ADCY3 plays an important role in the regulation of adiposity and glucose homeostasis. Similar to one of the possible mechanism of PON2, obesity is a risk factor for many metabolic diseases and even cancer [35].

Many studies on PCSK9 variants have demonstrated that it was related to plasma lipid, especially cholesterol metabolism [36,37]. Cholesterol accumulation in the liver is an early event in NAFLD, and the whole-body cholesterol homeostasis is mainly maintained by the liver [37]. PCSK9 is an enzyme that binds to LDLR and promotes its degradation in the lysosomal pathway, inhibition of PCSK9 prevents LDLR degradation, resulting in increased availability of LDLR to facilitate LDL clearance38. It was found that PCSK9 can bind to LDLR and promote its degradation through lysosomal pathway, and inhibition of PCSK9 can prevent LDLR degradation, thereby increasing the availability of LDLR and promoting LDL clearance [38]. This could further reduce the risk of NAFLD, but the specific mechanism still needs to be confirmed further.

GCKR variations have been shown to be associated with NAFLD [25,26]. Loss-of-function GCKR mutation (rs1260326), encoding for the P446L protein variant, seems to represent the causal variant underlying the association with hepatic fat accumulation [39]. This process occurs mainly by increasing the production of malonyl-CoA, which is a fatty substrate that prevents the oxidation of fatty acids, leading to the accumulation of fat in the liver [26].

Multiple environmental risk factors, including age, gender, race, ethnicity, obesity metabolic conditions, chronic infections, and other conditions are associated with fatty liver. In the present study, demographic characteristics and lifestyle factors of the participants, including waistline, gender, dyslipidemia, hypertension, sweet consumption, egg consumption, and vegetable consumption influenced NAFLD. This has been confirmed in previous studies [3,7,8,9,10,39,40,41].

There is no doubt that NAFLD is the result of a combination of genetic and lifestyle factors, and there are complex interactions between them. As is well known, lifestyle factors themselves are partly determined by genetic factors. In addition, increasingly more evidence indicates that lifestyle can also alter the impact of genetic variations on NAFLD [41,42]. The reasons for this, though, are not particularly clear. Maybe it is because they share etiological pathways. Therefore, we explored the interactions of gene biochemical indicators, gene lifestyle factors, and certain lifestyle factors with the risk of NAFLD. Our findings indicate that compared with individuals with similar lifestyle but lower genetic risk, individuals with high genetic risk have a higher starting risk of developing NAFLD. Interactions between some variables were found, suggesting that the estimated joint effect was greater than the sum of the estimated effects of either alone. However, due to the small sample size, the interpretation of these results should be cautious. If the conclusion is to be confirmed, independent studies with a larger sample size should be repeated.

This study had some other limitations. First, our model was designed to explain the relationship between variables and disease and not to predict the risk of NAFLD, and thus the model was not tested in new populations, which means that conclusions from this study can only infer association rather than causation. Second, most responses related to lifestyles were obtained through questioning of the patients, and thus may be influenced by recall bias and social desirability. Moreover, the conclusions may only be applicable to people in southern China. Studies in multiple regions and different populations using a randomized, large-scale, long-term design are needed.

## 5. Conclusions

In conclusion, we identified three SNPs and six environmental factors associated with NAFLD. These results provide the theoretical basis for gene and other risk factors screening to prevent NAFLD.

## Figures and Tables

**Table 1:** Basic characteristics.

Characteristic	NAFLD (+)	NAFLD (−)	t/z/χ^2^	*p*
Total	327	1836		
Age, Median (IQR), year	64 (14.50)	62 (17.00)	0.13	0.717
Sex, *n* (%)			3.02	0.082
Male	49 (49.00)	917 (45.50)		
Female	51 (51.00)	1096 (54.5)		
Weight, Median (IQR), year	63.2 (5.05)	59.4 (12.82)	158.93	<0.001
BMI, mean ± SD, kg/m^2^	24.06 (4.55)	23.15 (3.84)	194.48	<0.001
Waistline, Median (IQR), cm	82.87 (11.00)		126.40	<0.001
SBP, Median (IQR), mmHg	145 (16.50)	136 (30.00)	16.62	<0.001
DBP, Median (IQR), mmHg	90 (15.00)	80 (16.00)	17.27	<0.001
TC, Median (IQR), mmol/L	4.99 (1.39)	4.85 (1.27)	28.68	<0.001
TG, Median (IQR), mmol/L	1.50 (0.65)	1.28 (0.87)	101.84	<0.001
HDL-C, Median (IQR), mmol/L	1.24 (0.32)	1.26 (0.38)	33.34	<0.001
LDL-C, Median (IQR), mmol/L	3.18 (1.37)	2.98 (1.11)	5.11	0.0238
The history of diabetes				0.0082
+	23 (7.00)	70 (3.80)	7.00	
−	304 (93.00)	1766 (96.20)		
The intake of vegetables, *n* (%)			16.63	0.0023
<45 g/day	107 (32.70)	1499 (81.60)		
≥45 g/day	220 (67.30)	337 (18.40)		
The intake of egg, *n* (%)			18.46	0.0004
<4 eggs/week	86 (26.30)	1560 (85.00)		
≥4 eggs/week	241 (73.70)	276 (15.00)		
The intake of fried, *n* (%)			34.82	<0.001
Never	175 (53.50)	679 (37.00)		
Regularly	152 (46.50)	1157 (63.00)		
The intake of sweet, *n* (%)			55.58	<0.001
<4 times/week	177 (54.10)	1715 (93.40)		
≥4 times/week	150 (45.90)	121 (6.60)		

NAFLD, non-alcoholic fatty liver disease; SBP, systolic blood pressure; DBP, diastolic blood pressure; TC, total cholesterol; TG, triglycerides; IQR, interquartile range; SD, standard deviation; BMI, body mass index; HDL-C, high-density lipoprotein-cholesterol; LDL-C, low-density lipoprotein cholesterol; t, the value of t test; z, the value of Wilcoxon rank sum test; χ2, the value of hi-squared test. *p*-value < 0.05 are considered statistically significant and maintains significance using the Benjamini–Hochberg procedure with the false discovery rate at 0.09.

**Table 2:** Associations of genetic models with risk of non-alcoholic fatty liver disease (NAFLD).

SNP	Genotype	Unadjusted OR (95%CI)	Unadjusted *p*	Adjusted OR (95%CI)	Adjusted *p*
rs7493					
additive	CG/CC	1.27 (0.98–1.64)	0.065	1.26 (0.97–1.62)	0.088
	GG/CC	1.82 (0.10–3.29)	0.049	1.87 (1.01–3.48)	0.046
dominant	GG + CG/CC	1.31 (1.03–1.68)	0.026	1.31 (1.01–1.69)	0.036
recessive	GG/CG + CC	1.68 (0.93–3.03)	0.083	1.75 (0.95–3.22)	0.074
rs7593130					
additive	CT/TT	1.03 (0.79–1.35)	0.800	0.95 (0.72–1.25)	0.706
	CC/TT	1.57 (1.12–2.19)	0.009	1.46 (1.04–2.07)	0.031
dominant	CC + CT/TT	1.15 (0.90–1.48)	0.252	1.07 (0.82–1.38)	0.626
recessive	CC/CT + TT	1.54 (1.14–2.07)	0.005	1.51 (1.11–2.05)	0.008
rs1260326					
additive	CT/CC	0.31 (0.11–0.84)	0.133	1.31 (0.93–1.84)	0.123
	TT/CC	1.48 (1.04–2.10)	0.031	1.48 (1.03–2.13)	0.033
dominant	TT + CT/CC	1.35 (0.99–1.86)	0.057	1.37 (0.99–1.90)	0.055
recessive	TT/CT + CC	1.23 (0.95–1.58)	0.112	1.22 (0.94–1.58)	0.133
rs11583680					
additive	CT/CC	0.63 (0.45–0.87)	0.005	0.61(0.424–086)	0.005
	TT/CC	0.35 (0.08–1.49)	0.156	0.47 (0.11–2.06)	0.311
dominant	TT + CT/CC	0.61 (0.44–0.83)	0.002	0.61 (0.44–0.85)	0.003
recessive	TT/CT + CC	0.38 (0.09–1.62)	0.19	0.37 (0.12–2.20)	0.368

Adjust factors: dyslipidemia, sex, hypertension, the intake of egg, the intake of sweets, and the intake of vegetables. *p*-value < 0.05 was considered statistically significant.

**Table 3:** Associations of gene score and lifestyles with risk of NAFLD.

Characters	OR	95%CI	*p*
gene score	1.49	1.23–1.81	<0.001
Dyslipidemia	2.42	1.84–3.19	<0.001
Sex	1.24	0.98–1.57	0.083
The intake of egg	6.52	5.23–8.11	<0.001
Hypertension	1.02	1.01–1.02	<0.001
The intake of sweet	4.36	3.51–5.41	<0.001
The intake of vegetable	0.29	0.24–0.35	<0.001

The intake of egg classified into <4 eggs every week, ≥4 eggs every week. The intake of sweet classified into <4 times every week, ≥4 times every week. The intake of vegetable classified into <45 g every day, ≥45 g every day; *p*-value < 0.05 was considered statistically significant.

**Table 4:** Interactions between gene polymorphism and the intake of vegetables for the risk of NAFLD.

SNPs	Adequate Vegetables		Inadequate Vegetables		OR (95%CI) for Hypertension Patients within Strata of Genotype	RERI (95%CI)	*p*
	Case/Control (*n*)	OR (95%CI)	Case/Control (*n*)	OR (95%CI)			
rs11583680							
Non-risk allele carriers (TT + CT)	20/348		30/72				
		1		7.25 (3.90–13.48)	7.25 (3.90–13.48)		
				*p* < 0.001	*p* < 0.001		
Risk allele carriers (CC)	87/1151		190/265			4.91 (0.66–9.17)	0.024
		1.32 (0.80–2.17)		12.99 (8.77–19.22)	9.49 (7.12–12.64)		
		*p* = 0.283		*p* < 0.001	*p* < 0.001		
OR (95%CI) for risk allele carriers within strata of vegetable intake		1.32 (0.80–2.17)		1.31 (1.04–1.66)			
		*p* = 0.283		*p* = 0.022			
						
rs7593130							
Non-risk allele carriers (CT + TT)	85/1272		174/296				
		1		8.80 (6.59–11.74)	8.80 (6.59–11.74)		
				*p* < 0.001	*p* < 0.001		
Risk allele carriers (CC)	22/227		46/41			7.55 (0.13–14.97)	0.046
		1.45 (0.90–2.37)		16.79 (10.44–26.99)	11.58 (6.31–21.25)		
		*p* = 0.137		*p* < 0.001	*p* < 0.001		
OR (95%CI) for risk allele carriers within strata of vegetable intake		1.45 (0.90–2.37)		1.38 (1.10–1.74)			
		*p* = 0.137		*p* = 0.006			
						
rs7493							
Non-risk allele carriers (CC)	62/1022		142/238				
		1		9.84 (7.07–13.68)	9.84 (7.07–13.68)		
				*p* < 0.001	*p* < 0.001		
Risk allele carriers (GG + CG)	45/477		78/99			2.60 (−1.81–7.00)	0.248
		1.56 (1.04–2.32)		12.99 (8.77–19.22)	8.35 (5.46–12.79)		
		*p* = 0.03		*p* < 0.001	*p* < 0.001		
OR (95%CI) for risk allele carriers within strata of vegetable intake		1.56 (1.04–2.32) *p* = 0.03		1.15 (0.96–1.38) *p* = 0.133			

RERI, relative excess risk due to interaction; *p*-value < 0.05 is considered statistically significant.

**Table 5:** Interactions between dyslipidemia and hypertension/other lifestyles for the risk of NAFLD.

Other Lifestyles	Dyslipidemia (−)		Dyslipidemia (+)		OR (95%CI) for Hypertension Patients within Strata of Genotype	RERI (95%CI)	*p*
	Case/Control (*n*)	OR (95%CI)	Case/Control (*n*)	OR (95%CI)			
Hypertension							
Hypertension (−)	33/459		90/423				
		1		2.96 (1.94–4.51)	2.96 (1.94–4.51)		
				*p* < 0.001	*p* < 0.001		
Hypertension (+)	41/302		163/652			−0.37 (−1.56–0.82)	0.541
		1.89 (1.17–3.05)		3.48 (2.35–5.15)	1.84 (1.27–2.66)		
		*p* = 0.01		*p* < 0.001	*p* = 0.001		
OR (95%CI) for risk allele carriers within strata of dyslipidemia		1.89 (1.17–3.05)		1.08 (0.94–1.25)			
		*p* = 0.01		*p* = 0.267			
						
The intake of vegetables							
Adequately	19/613		88/886				
		1		3.20 (1.93–5.32)	3.20 (1.93–5.32)		
				*p* < 0.001	*p* < 0.001		
Inadequately	55/148		165/189			13.97 (4.81–23.12)	0.002
		11.99 (6.91–20.81)		28.17 (17.05–46.53)	2.35 (1.62–3.41)		
		*p* < 0.001		*p* < 0.001	*p* < 0.001		
OR (95%CI) for risk allele carriers within strata of dyslipidemia		11.99 (6.91–20.81)		2.96 (2.55–3.45)			
		*p* < 0.001		*p* < 0.001			
						
The intake of egg							
Adequately	18/658		68/902				
		1		2.76 (1.62–4.68)	2.76 (1.62–4.68)		
				*p* < 0.001	*p* < 0.001		
Inadequately	56/103		185/173			17.47 (4.55–30.39)	0.008
		19.88 (11.24–35.12)		39.09 (24.43–65.23)	1.97 (1.34–2.89)		
		*p* < 0.001		*p* < 0.001	*p* = 0.001		
OR (95%CI) for risk allele carriers within strata of dyslipidemia		11.99 (6.91–20.81) *p* < 0.001		3.77 (3.20–4.24) *p* < 0.001			
The intake of sweetmeat							
Adequately	10/615		41/690				
		1		2.38 (1.66–3.42)	2.38 (1.66–3.42)		
				*p* < 0.001	*p* < 0.001		
Inadequately	10/370		32/395			12.08 (1.77–22.38)	0.022
		13.12 (7.57–22.74)		26.57 (17.35–40.70)	2.03 (1.19–3.46)		
		*p* < 0.001		*p* < 0.001	*p* = 0.010		
OR (95%CI) for risk allele carriers within strata of dyslipidemia		1.66 (0.69–4.03) *p* = 0.261		3.34 (2.82–3.96) *p* < 0.001			

*p*-value < 0.05 is considered statistically significant.

## Supplementary Materials

The following are available online at https://www.mdpi.com/1660-4601/17/14/5217/s1, Table S1: The information of 107 SNPs. Table S2: Elastic net regularization feature selection for gene score and lifestyles. Table S3: Interactions between gene polymorphism and the intake of egg for the risk of NAFLD. Table S4: Interactions between gene polymorphism and the intake of dessert for the risk of NAFLD. Table S5: Interactions between gene polymorphism and hypertension for the risk of NAFLD. Table S6: Interactions between gene polymorphism and dyslipidemia for the NAFLD.
