# Supplementary material for: Association of Genetic and Environmental Factors with Non-Alcoholic Fatty Liver Disease in a Chinese Han Population
Source: Int J Environ Res Public Health. 2020 Jul 20;17(14):5217. doi: 10.3390/ijerph17145217 (PMC7399983; doi:10.3390/ijerph17145217)
Supplement: Supplementary file 1 [file ijerph-17-05217-s001.pdf]

Supplementary Materials:

Table S1. The information of 107 SNPs.

SNP	Gene	Chromosome	Location	Minor Gene	MAF
rs629301	CELSR2	1	109818306	A	G = 0.2390
rs10889353	DOCK7	1	63118196	A	C = 0.3311
rs4846914	GALNT2	1	230295691	G	A = 0.3339
rs111563724	PCSK9	1	55527062	T	NA
rs11583680	PCSK9	1	55505668	C	T = 0.0913
rs17111503	PCSK9	1	55503448	A	G = 0.2969
rs2479409	PCSK9	1	55504650	A	G = 0.3986
rs2483205	PCSK9	1	55518316	C	T = 0.3942
rs662145	PCSK9	1	55529828	C	C = 0.2758
rs12130333	ANGPTL3	1	63191777	C	T = 0.1256
rs4299376	ABCG8	2	44072576	T	G = 0.1705
rs10187348	ADCY3	2	25078415	G	C = 0.3347
rs1127568	ADCY3	2	25046090	G	T = 0.3087
rs11689546	ADCY3	2	25130451	A	A = 0.4651
rs1344840	ADCY3	2	25070645	C	A = 0.1360
rs2241759	ADCY3	2	25064193	A	G = 0.4894
rs2278485	ADCY3	2	25057263	G	A = 0.4121
rs4077678	ADCY3	2	25122840	G	C = 0.4509
rs4665273	ADCY3	2	25046355	C	T = 0.4273
rs6751537	ADCY3	2	25062320	A	G = 0.3690
rs7593130	ADCY3	2	25138987	T	C = 0.3516
rs7604576	ADCY3	2	25047408	A	A = 0.4784
rs7608976	ADCY3	2	25075281	A	G = 0.4453
rs693	APOB	2	21232195	C	A = 0.2510
rs1260326	GCKR	2	27730940	C	T = 0.2933
rs515135	APOB	2	21286057	G	T = 0.2494
rs1229984	ADH1B	4	100239319	G	T = 0.1585
rs12654264	HMGCR	5	74648603	T	T = 0.4357
rs11759908	TFEB	6	41702979	C	T = 0.2778
rs6939861	TFEB	6	41703041	G	A = 0.2606
rs3812316	MLXIPL	7	73020337	C	G = 0.0683
rs7493	PON2	7	95034775	C	C = 0.2833
rs17145738	TBL2	7	72982874	C	T = 0.0821
rs17321515	NearTRIB1	8	126486409	A	G = 0.4798
rs2954029	Near TRIB1	8	126490972	A	T = 0.4050
rs328	LPL	8	19819724	C	G = 0.0925
rs10820743	ABCA1	9	107671659	T	C = 0.3135
rs11789818	ABCA1	9	107547230	C	T = 0.2714
rs2000069	ABCA1	9	107635869	T	C = 0.4313
rs2065412	ABCA1	9	107598740	T	C = 0.1859
rs2254884	ABCA1	9	107581749	A	C = 0.2833
rs2297406	ABCA1	9	107551538	C	T = 0.2236
rs2472377	ABCA1	9	107687104	A	C = 0.4826
rs2472386	ABCA1	9	107601541	T	G = 0.4285
rs2472433	ABCA1	9	107623326	A	T = 0.4397
rs2472510	ABCA1	9	107683122	A	G = 0.2588
rs2482433	ABCA1	9	107546901	A	G = 0.1340
rs2515614	ABCA1	9	107684318	T	C = 0.3079
rs2515616	ABCA1	9	107681995	C	A = 0.1845
rs2515617	ABCA1	9	107680915	C	A = 0.4385
rs2575876	ABCA1	9	107665739	G	A = 0.2632
rs2740479	ABCA1	9	107563437	C	A = 0.3964
rs2740484	ABCA1	9	107551180	G	T = 0.1721

rs2740486	ABCA1	9	107666513	C	T = 0.4004
rs4149264	ABCA1	9	107677211	C	C = 0.1613
rs4149336	ABCA1	9	107550639	T	G = 0.3061
rs4149339	ABCA1	9	107545156	T	A = 0.4411
rs4743763	ABCA1	9	107593182	T	A = 0.4525
rs4743764	ABCA1	9	107629104	C	T = 0.4623
rs6479282	ABCA1	9	107601512	T	C = 0.4060
rs4149269	ABCA1	9	107647121	C	G = 0.4499
rs10431036	BCO2	11	112047061	G	A = 0.1478
rs11214109	BCO2	11	112044226	C	T = 0.1560
rs12420476	BCO2	11	112044398	A	C = 0.0755
rs13328843	BCO2	11	112044687	G	C = 0.4712
rs174547	FADS1	11	61570783	C	C = 0.2979
rs1148058	KCNJ1	11	128712781	G	A = 0.1779
rs2846679	KCNJ1	11	128729544	G	A = 0.1148
rs675388	KCNJ1	11	128708009	T	A = 0.1573
rs675759	KCNJ1	11	128707975	C	C = 0.1643
rs964184	ZPR1	11	116648917	C	G = 0.2222
rs671	ALDH2	12	112241766	G	A = 0.0357
rs2228576	SCNN1A	12	6457062	G	T = 0.2602
rs3782724	SCNN1A	12	6466081	G	G = 0.2522
rs2650000	Near HNF1A	12	121388962	G	A = 0.3087
rs17845226	ANXA2	15	60653205	C	A = 0.0485
rs1800588	LIPC	15	58723675	C	T = 0.3864
rs12438818	SLC12A1	15	48596166	T	A = 0.1793
rs8025278	SLC12A1	15	48595192	T	G = 0.2648
rs11641677	BCMO1	16	81271729	A	G = 0.4056
rs11646692	BCMO1	16	81271906	C	G = 0.3578
rs12934922	BCMO1	16	81301694	A	T = 0.2272
rs6564851	BCMO1	16	81264597	T	T = 0.4762
rs7501331	BCMO1	16	81314496	C	T = 0.1522
rs3764261	CETP	16	56993324	G	A = 0.2895
rs16942887	PSKH1	16	67928042	G	A = 0.1522
rs7205273	SCNN1B	16	23324849	C	T = 0.2320
rs7200183	SCNN1G	16	23206608	C	C = 0.3988
rs11643718	SLC12A3	16	56933519	G	A = 0.0799
rs3812963	SLC12A3	16	56948991	G	T = 0.1448
rs4784733	SLC12A3	16	56899006	C	C = 0.2837
rs5805	SLC12A3	16	56947522	C	A = 0.4087
rs4939883	Near LIPG	18	47167214	T	T = 0.2498
rs7241918	Near LIPG	18	47160953	T	G = 0.1004
rs4420638	APOC1	19	45422946	G	G = 0.1510
rs5167	APOC2	19	45448465	G	G = 0.4393
rs16996148	CILP2	19	19658472	G	T = 0.1156
rs1003723	LDLR	19	11224181	C	T = 0.2768
rs2738466	LDLR	19	11242765	G	G = 0.2344
rs5929	LDLR	19	11226800	C	T = 0.1218
rs6413504	LDLR	19	11241915	A	G = 0.3510
rs10401969	SUGP1	19	19407718	T	C = 0.1176
rs1800961	HNF4A	20	43042364	C	T = 0.0240

MAF:Minimum allele frequency.

Table S2. Elastic net regularisation feature selection for gene-score and lifestyles.

Elastic Net Regularisation Feature Selection/Model	Variable Weighting/Cofe
gene-score	0.451
Dyslipidemia	0.317
Sex	0.252
The intake of egg	1.490
Hypertension	0.236
The intake of sweet	−0.350
The intake of vegetable	−0.939

Table S3. Interactions between Gene polymorphism and the intake of egg for the risk of NAFLD.

	**1–4 Eggs/Week**		**More than 4 Eggs/Week**		**OR (95%CI) for Hypertension Patients within Strata of Genotype**	**RERI (95%CI)**	***p***
	**Case/Control (*n*)**	**OR (95%CI）**	**Case/Control (*n*)**	**OR (95%CI)**			
rs11583680							
Non-risk allele carriers (TT + CT)	10/350	1	40/70	20.00 (9.55–41.87) ***p* < 0.001**	20.00 (9.55–41.87) ***p* < 0.001**		
Risk allele carriers (CC)	76/1210	2.20 (1.13–4.30) ***p* = 0.021**	201/206	34.15 (17.69–65.94) ***p* < 0.001**	15.54 (11.48–21.02) ***p* < 0.001**	4.91 (−0.76–13.17)	0.064
OR (95%CI) for risk allele carriers within strata of egg intake		2.20 (1.13–4.30) ***p* = 0.021**		1.71 (1.11–2.64) ***p* = 0.016**			
rs7593130							
Non-risk allele carriers (CT + TT)	65/1325	1	194/243	16.27 (11.91–22.24) ***p* < 0.001**	16.27 (11.91–22.24) ***p* < 0.001**		
Risk allele carriers (CC)	21/235	1.82 (1.09–3.04) ***p* = 0.021**	47/33	29.03 (17.43–48.35) ***p* < 0.001**	15.94 (8.49–29.94) ***p* < 0.001**	11.92 (−1.65–25.48)	0.085
OR (95%CI)for risk allele carriers within		1.82 (1.09–3.04) ***p* = 0.021**		1.34 (1.05–1.70) ***p* = 0.019**			

strata of egg intake							
rs7493							
Non-risk allele carriers (CC)	55/1070	1	149/190	15.26 (10.80–21.56) **$p < 0.001$**	15.26 (10.80–21.56) **$p < 0.001$**		
Risk allele carriers (GG + CG)	31/490	1.23 (0.78–1.94) $p = 0.369$	92/86	12.99 (8.77–19.22) **$p < 0.001$**	16.91 (10.60–26.98) **$p < 0.001$**	5.33 (−1.72–12.39)	0.138
OR (95%CI) for risk allele carriers within strata of egg intake		1.23 (0.78–1.94) $p = 0.369$		1.17 (0.97–1.40) $p = 0.094$			

Bold values are statistically with *p*-value < 0.05

Table S4 Interactions between Gene polymorphism and the intake of dessert for the risk of NAFLD

	1–4 times/week		More than 4 times/week		OR (95%CI) for Hypertension Patients within Strata of Genotype	RERI (95%CI)	*p*
	**Case/Control (n)**	**OR (95%CI）**	**Case/Control (n)**	**OR (95%CI）**			
rs11583680							
Non-risk allele carriers (TT + CT)	30/401	1	20/19	14.07 (6.79–29.18) ***p* < 0.001**	14.07 (6.79–29.18) ***p* < 0.001**		
Risk allele carriers (CC)	147/1314	1.50 (0.99–2.25) *p* = 0.053	130/102	17.04 (10.83–26.79) ***p* < 0.001**	11.39 (8.35–15.54) ***p* < 0.001**	6.43 (−1.85–14.72)	0.128
OR(95%CI) for risk allele carriers within **strata of** dessert intake		1.50 (0.99–2.25) *p* = 0.053		1.10 (0.78–1.55) *p* = 0.581			
rs7593130							
Non-risk allele carriers (CT + TT)	14/1457	1	118/111	10.99 (8.05–15.00) ***p* < 0.001**	10.99 (8.05–15.00) ***p* < 0.001**		
Risk allele carriers (CC)	36/258	1.44 (0.93–2.25) *p* = 0.065	32/10	33.07 (15.92–68.67) ***p* < 0.001**	22.93 (10.40–50.59) ***p* < 0.001**	21.65 (−2.30–45.60)	0.076
OR (95%CI) for risk allele carriers within **strata of** dessert intake		1.44 (0.93–2.25) *p* = 0.065		1.74 (1.19–2.53) ***p* = 0.004**			
rs7493							
Non-risk allele carriers	112/1174		92/86				

(CC)		1		11.21 (7.89–15.95)<br>**p < 0.001**	11.21 (7.89–15.95)<br>**p < 0.001**		
Risk allele carriers<br>(GG + CG)	65/541	1.26 (0.91–1.74)<br>p = 0.161	58/35	17.37 (10.94–27.58)<br>**p < 0.001**	13.79 (8.43–22.56)<br>**p < 0.001**	2.47 (−7.43–12.36)	0.625
OR (95%CI) for risk allele carriers within **strata of** dessert intake		1.26 (0.91–1.74)<br>p = 0.161		1.25(0.96–1.61)<br>p = 0.094			

Bold values are statistically with p-value < 0.05

Table S5. Interactions between Gene polymorphism and hypertension for the risk of NAFLD.

	Hypertension (−)		Hypertension (+)		OR (95%CI) for Hypertension Patients within Strata of Genotype	RERI (95%CI)	*p*
	Case/Control (n)	OR (95%CI）	Case/Control (n)	OR (95%CI）			
rs**11583680**							
Non-risk allele carriers (TT + CT)	22/203		28/217				
		1		1.19 (0.66–2.15)<br>*p* = 0.562	1.19 (0.66–2.15)<br>*p* = 0.562		
Risk allele carriers (CC)	101/679		176/7376			0.64 (−0.04–1.32)	0.064
		1.37 (0.84–2.23)<br>*p* = 0.203		2.20 (1.38–3.52)<br>***p* = 0.001**	1.65 (1.23–2.09)<br>***p* < 0.001**		
OR(95%CI) for risk allele carriers within **strata of hypertension**		1.37 (0.84–2.23)<br>*p* = 0.203		1.36 (1.10–1.68)<br>***p* = 0.005**			
rs**7593130**							
Non-risk allele carriers (CT + TT)	94/746		165/8227				
		1		1.59 (1.21–2.09)<br>***p* = 0.001**	1.59 (1.21–2.09)<br>***p* = 0.001**		
Risk allele carriers (CC)	29/136		39/132			−0.06 (−1.07–1.19)	0.92
		1.69 (1.07–2.67)<br>***p* = 0.023**		2.34 (1.55–3.56)<br>***p* < 0.001**	1.38 (0.81–2.37)<br>*p* = 0.234		
OR (95%CI) for risk allele carriers within **strata of hypertension**		1.69 (1.07–2.67)<br>***p* = 0.023**		1.21(0.99–1.48)<br>*p* = 0.055			
rs7493							
Non-risk allele carriers (CC)	76/606		128/654				

		1		1.56 (1.15–2.12) **p = 0.004**	1.56 (1.15–2.12) **p = 0.004**		
Risk allele carriers (GG + CG)	47/276		76/300			0.74 (−0.47–1.95)	0.792
		1.35 (0.92–2.01) p = 0.125		4.98 (2.42–10.25) **p < 0.001**	1.49 (1.00–2.21) **p = 0.05**		
OR (95%CI) for risk allele carriers within **strata of hypertension**		1.35 (0.92–2.01) p = 0.125		1.14 (0.97–1.33) p = 0.108			

Bold values are statistically with p-value < 0.05

Table S6. Interactions between Gene polymorphism and dyslipidemia for the NAFLD.

	Dyslipidemia (−)		Dyslipidemia (+)		OR (95%CI) for Hypertension Patients within Strata of Genotype	RERI (95%CI)	*p*
	**Case/Control (n)**	**OR (95%CI)**	**Case/Control (n)**	**OR (95%CI)**			
rs11583680							
Non-risk allele carriers (TT + CT)	11/181	1	39/239	2.69 (1.34–5.39) *p* = 0.005	2.69 (1.34–5.39) *p* = 0.005		
Risk allele carriers (CC)	63/580	1.79 (0.92–3.46) *p* = 0.085	214/836	4.21 (2.25–7.88) *p* < 0.001	2.36 (1.75–3.18) *p* < 0.001	0.74 (−1.37–1.78)	0.231
OR(95%CI)for risk allele carriers within strata of dyslipidemia		1.79 (0.92–3.46) *p* = 0.085		1.25(1.04–1.51) *p* = 0.017			
rs7593130							
Non-risk allele carriers (CT + TT)	56/651	1	203/917	2.57 (1.88–3.52) *p* < 0.001	2.57 (1.88–3.52) *p* < 0.001		
Risk allele carriers (CC)	18/110	1.90 (1.08–3.36) *p* = 0.027	50/158	3.68 (2.42–5.59) *p* < 0.001	1.93 (1.07–3.49) *p* = 0.029	0.25(−1.37–1.78)	0.798
OR (95%CI) for risk allele carriers within strata of dyslipidemia		1.90 (1.08–3.36) *p* = 0.027		1.20 (1.00–1.43) *p* = 0.047			
rs7493							
Non-risk allele carriers (CC)	46/514		158/746				

		1		2.37 (1.67–3.35)<br>$p < 0.001$	2.37 (1.67–3.35)<br>$p < 0.001$		
Risk allele carriers (GG + CG)	28/247		95/329			0.59(−0.39–1.57)	0.237
		1.27 (0.77–2.08)<br>$p = 0.348$		3.23 (2.21–4.71)<br>$p < 0.001$	2.55 (1.62–4.01)<br>$p < 0.001$		
OR (95%CI) for risk allele carriers within strata of dyslipidemia		1.27 (0.77–2.08)<br>$p = 0.348$		1.17(1.01–1.35)<br>$p = 0.033$			

Bold values are statistically with p-value < 0.05.
